# Serum Samples That Have Been Stored Long-Term (>10 Years) Can Be Used as a Suitable Data Source for Developing Cardiovascular Risk Prediction Models in Large Observational Rheumatoid Arthritis Cohorts

**DOI:** 10.1155/2014/930925

**Published:** 2014-09-11

**Authors:** Elke E. A. Arts, Calin D. Popa, Jacqueline P. Smith, Onno J. Arntz, Fons A. van de Loo, Rogier Donders, Anne Grete P. Semb, George D. Kitas, Piet L. C. M. van Riel, Jaap Fransen

**Affiliations:** ^1^Department of Rheumatology, Radboud University Medical Centre, Nijmegen, The Netherlands; ^2^Department of Rheumatology, Dudley Group NHS Foundation Trust, Dudley, UK; ^3^Department of Epidemiology, Biostatistics and Health Technology Assessment, Radboud University Medical Centre, Nijmegen, The Netherlands; ^4^Department of Rheumatology, Diakonhjemmet Hospital, Oslo, Norway

## Abstract

*Objective*. There is an unmet need for a specific cardiovascular risk (CV) algorithm for rheumatoid arthritis (RA) patients. Lipoprotein data are often not available in RA cohorts but could be obtained from frozen blood samples. The objective of this study was to estimate the storage effect on lipoproteins in long-term (>10 years) frozen serum samples. *Methods*. Data were used from an inception RA cohort. Multiple serum samples from 152 patients were analyzed for lipoproteins, being frozen for 1–26 years at −20°C. Storage effect on lipoproteins was estimated using longitudinal regression analyses and a lipid decay correction factor was developed. Clinical impact of the storage effect on lipoproteins was assessed by calculating the number of patients reclassified to another CV risk group according to the SCORE risk calculator after applying the decay correction factor. *Results*. There was a significant effect of storage time on total cholesterol (TC) (*P* < 0.001) and high density lipoprotein cholesterol (HDL-c) levels (*P* < 0.001), not LDL-c (*P* = 0.83). The lipid decay correction factor was 0.03 mmol/L and 0.024 mmol/L per additional year of storage for TC and HDL-c, respectively. The TC : HDL ratio decreased after correction for storage effect. After correction, only 5% of patients were reclassified to another CV risk group. *Conclusion*. A modest storage decay effect on lipoproteins was found that is unlikely to significantly affect CV risk stratification. Serum samples that have been stored long-term (>10 years) can be used to obtain valid lipid levels for developing CV risk prediction models in RA cohorts, even without applying a decay correction factor.

## 1. Introduction

Cardiovascular (CV) risk is increased in patients with rheumatoid arthritis (RA) [1]. Cardiovascular diseases account for 50% of all excess mortality in RA patients [1]. RA itself as a chronic inflammatory condition may increase CV risk. Also, studies have shown that inflammation may modulate traditional CV risk factors [2, 3]. Atherosclerotic plaques in the carotid artery appear more severe and prevalent in RA patients compared to the general population [4–7]. In comparison to healthy controls and RA patients in remission, RA patients with active disease seem to have less stable plaques that are more vulnerable to rupture, which increases the probability of a CV event. Considering the increased risk of CV disease, prevention of CV disease is important. According to international guidelines this includes adequate risk assessment using a CV risk algorithm. In the general population, several algorithms for the prediction of CV risk are available, such as the systematic coronary risk evaluation (SCORE) and the Framingham risk score [9, 10]. It has recently been reported that both the SCORE and Framingham risk score algorithm provide suboptimal CV risk estimates in patients with RA [11, 12]. To improve CV risk assessment in RA patients, disease specific risk factors may be required such as RA disease activity. Also, other cardiovascular-related parameters not incorporated in the present algorithms, such as carotid artery intima-media index (cIMT), the presence of plaques in these patients, or certain genetic markers associated with CV risk factors, might also be taken into consideration [13–17]. There is an unmet need for development of a RA specific CV risk calculator.

In order to develop and validate appropriate CV risk models for patients with RA, it would be advantageous to use data from existing cohorts with long follow-up. In existing cohorts of RA patients, lipid levels usually are not determined at baseline. Long-term storage may lead to degradation of cholesterol, that is, a lipid decay. Consequently, if lipids were to be measured in samples that have been stored for longer periods of time, cholesterol levels could be underestimated. Furthermore, if these lipid levels were used in CV risk algorithms, the result may be an underestimation of CV risk.

Within a timeframe of 1-2 years of storage, no change to moderate decreases in lipid levels have been reported [18–20], as well an increase of high density lipoprotein cholesterol (HDL-c) levels [21, 22]. Lipid decay seems to be smaller at lower temperatures [18–20]. This has led to the hypothesis that the HDL concentration influences the effect of storage on lipoproteins [23]. Overall, total cholesterol (TC) and triglyceride (TG) levels seems to decay less when stored than HDL-c levels [24, 25]. One study has investigated the effect of long-term storage on cholesterol levels for up to seven years of storage. A significant mean decrease of 2.0% per year storage in TC levels and a nonsignificant average 1.3% decrease per year storage in HDL-c levels were reported [26]. To our knowledge, the effect of longer storage (10 years) on serum cholesterol levels has not been investigated. Although deterioration of cholesterol content in stored serum samples can be expected, the magnitude of this effect after long periods of time is unknown. The objective of this study is to estimate the long time storage decay effect on TC and HDL-c levels in frozen serum samples of RA patients and to evaluate the clinical effect of the decay in CV risk models.

## 2. Methods

### 2.1. Study Design

**Figure 1:**
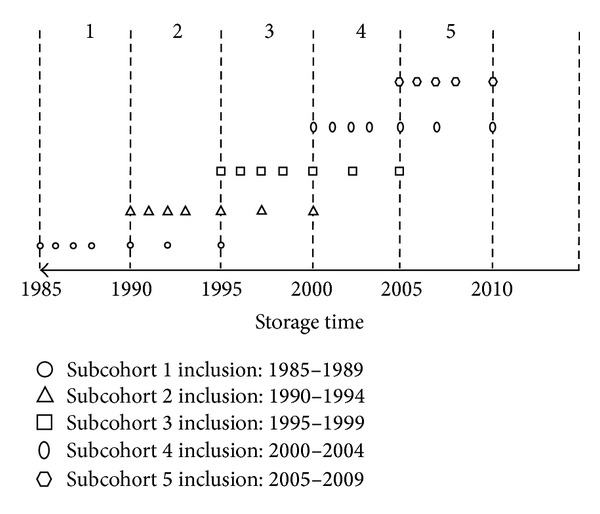
Storage and blood sampling times for the 5 subcohorts. Symbols represent the time points of the included selection of stored serum, during follow-up (baseline and year 1, 2, 3, 5, 7 and 10).

Serum samples at baseline and at 1, 2, 3, 5, 7, and 10 years of follow-up from patients included in the RA inception cohort of the Radboud University Nijmegen Medical Centre, from 1985 up to 2009 (*n* = 640), were used for measurements of lipoproteins. To test for a period effect, patients were stratified in five subcohorts according to year of inclusion in the cohort during 1985–1989, 1990–1994, 1995–1999, 2000–2004, and 2005–2009 (Figure 1). The study was approved by the Medical Ethical Committee and CMO Arnhem Nijmegen and informed consent was acquired from all participants.

### 2.2. Patients

Inclusion criteria for the early RA cohort were fulfillment of the 1987 ACR classification criteria for RA, disease duration less than one year, and being DMARD (disease-modifying antirheumatic drug) naïve. From this cohort, we selected at random 150 RA patients from the inception cohort using computer generated random numbers, to obtain 30 samples per subcohort.

### 2.3. Serum Samples

During follow-up, nonfasting blood samples were drawn annually by a trained nurse. Approximately 400 mL of serum was stored from each sample and divided into four separate vials. The samples were initially stored at −20°C. In 2008, all samples were transferred to storage facilities at −80°C. Blood samples collected from 2008 and thereafter were stored directly at −80°C. Blood samples obtained before 2007 were stored in 1.5 mL Eppendorf vials and samples obtained after 2007 were stored using Greiner “pp Cryovials.” Serum samples that were taken at baseline and during follow-up (at 1, 2, 3, 5, 7, and 10 years) were extracted from storage in January 2012. Immediately following this procedure, samples were prepared for cholesterol measurements and transported on dry ice to the laboratory facilities of Russells Hall Hospital, Dudley, UK.

### 2.4. Lipid Measurements

TC concentrations were measured enzymatically by means of the VITROS CHOL slide technique using the Triton X-100 surfactant, which is based on methods described previously [27]. HDL-c was measured using immunoturbidimetry. Low-density-lipoprotein cholesterol (LDL-c) was calculated using Friedewald's formula [28].

### 2.5. Statistical Analysis

The primary outcomes were the change in TC and HDL-c levels from baseline to after storage and the secondary outcome was the change in LDL-c level from baseline to after storage. To test for a period effect, a longitudinal regression analysis was used that corrects for repeated measurements within patients. Lipid level (TC and HDL-c) was the dependent variable and follow-up time; subcohort (1985–1989, 1990–1994, etc.) and an interaction term between follow-up time and subcohort were the main independent variables. As the course of cholesterol levels over follow-up time was nonlinear, a quadratic time term (time^2^) was included. To test for a period effect in the course of cholesterol levels, the interaction between subcohort and follow-up time was evaluated. Several variables were considered as potential confounders: age, gender, statin use at baseline, BMI, smoking, blood pressure, DAS28 score, rheumatoid factor positivity, and glucocorticosteroid use. Variables were considered confounders if their addition to the model led to a >10% change in one of the subcohort follow-up time effects.

For the development of a correction factor for the storage decay effect, linear mixed models were used, with cholesterol level as the dependent variable, storage time as primary independent variable, and the same confounders as in the analyses described previously. Storage time was calculated by subtracting the baseline date (date the blood samples were first frozen) from the date of serum analysis. The storage decay correction factor developed to adjust the TC, HDL-c, and LDL-c levels was defined as the estimated change in mmol/L cholesterol (*β*Chol) per additional unit of storage time (years) multiplied by the number of storage years (*t*) of a particular sample. When added to the measured cholesterol level (*β*observed), it gives an estimate of the “original” cholesterol value (*y*). The lipid storage decay factor is therefore *y* = *β*observed + (*β*Chol∗*t*).

In order to evaluate the clinical effect of the decay in CV risk models, reclassification across CV risk groups before and after correction was calculated. The SCORE risk algorithm was used to quantify the 10-year risk of CVD with and without correction for the storage decay effect. The CV risk was calculated with and without correction for the storage decay effect on lipids for all 1050 patients from the RA inception cohort. Reclassification of patients across CV risk groups (low <10%, intermediate 10–20%, and high <20%) was calculated. If CV risk of a patient exceeds 10%, primary prevention in the form of lifestyle changes or medical treatment is indicated according to European guidelines for CV prevention [29].

## 3. Results

### 3.1. Patients

One hundred and fifty two patients were included, evenly distributed across the 5 subcohorts (Table 1), with storage times ranging from 1 to 26 years. Samples from the oldest subcohorts comprised the longest storage times. Serum samples from seven time points (0, 1, 2, 3, 5, 7, and 10 years) were analyzed if available, yielding a total of 971 samples. Age, gender, rheumatoid factor (RF) positivity, disease activity index 28 joints (DAS28), use of statins, and glucocorticosteroids appeared to show trends over time (Table 1).

### 3.2. Differences between Subcohorts

**Figure 2:**
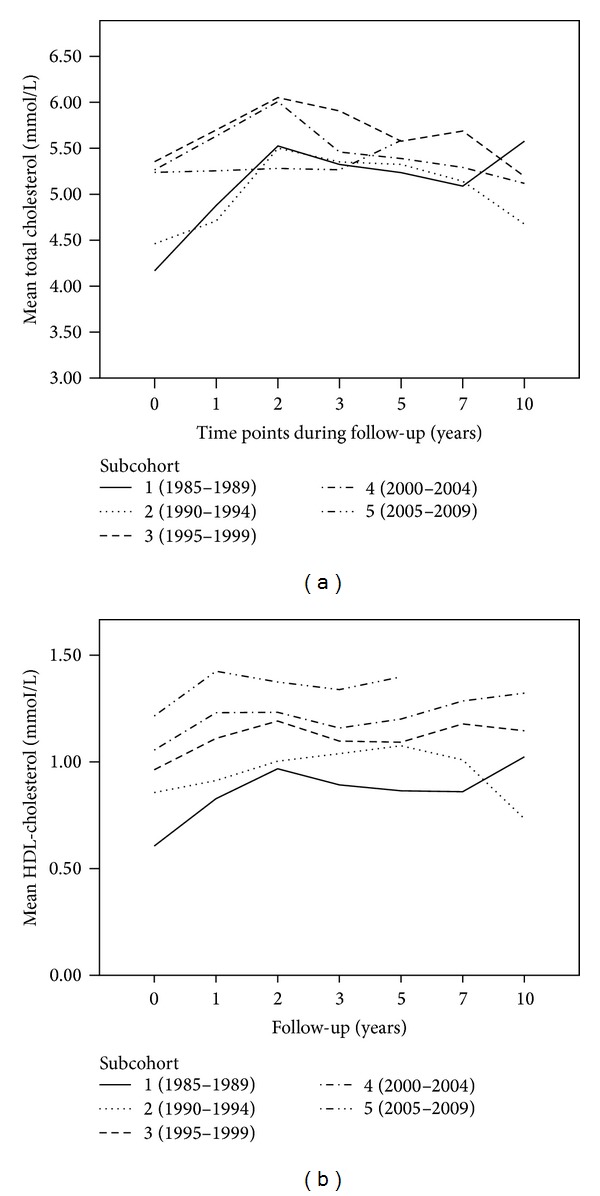
Lipoprotein levels measured in stored serum samples in the various subcohorts. Total cholesterol (a) and HDL-c (b) are depicted on the *y*-axis. Samples taken at the most recent follow-up moment in time, time point “10” on the *x*-axis, have the shortest follow-up time and samples taken at baseline (time point “0”) have been stored the longest.

Lipid levels measured at baseline are presented in Table 1. Lipid levels tended to be lowest in the subcohorts (Figure 2) that had the longest storage time and there appeared to be a nonlinear course of lipid levels during storage time. The unadjusted results (not shown) revealed a significant interaction effect between subcohort and follow-up time for TC and LDL-c (*P* = 0.02 and *P* = 0.01, resp.). Overall, the course of the various lipoprotein levels over time was not significantly different between subcohorts after adjustment for confounders (age, gender, and BMI) with *P* = 0.09, *P* = 0.05, and *P* = 0.18 for TC, LDL-c, and HDL-c, respectively. Rheumatoid factor and DAS28 were not confounders after these adjustments.

When looking specifically at the oldest and most recent cohort (the two extremes in terms of storage time), lipid levels in the oldest cohort were systematically lower than lipid levers in the most recent subcohort; a statistically significant difference for TC and LDL-c (*P* = 0.04 and *P* = 0.03) and a nonsignificant difference for HDL-c (*P* = 0.25) were found after correction for confounders.

### 3.3. Storage Time

There was a significant decay effect of storage time on TC and HDL-c levels (*P* < 0.001) (Tables 2 and 3). No effect of storage time was found for LDL-c levels (*P* = 0.83, data not shown). For the analysis for TC and LDL-c, age, gender, BMI, statin use, and glucocorticosteroid use at baseline were confounders and adjusted for. For HDL-c, a model with adjustment for age and gender sufficed.

**Figure 3:**
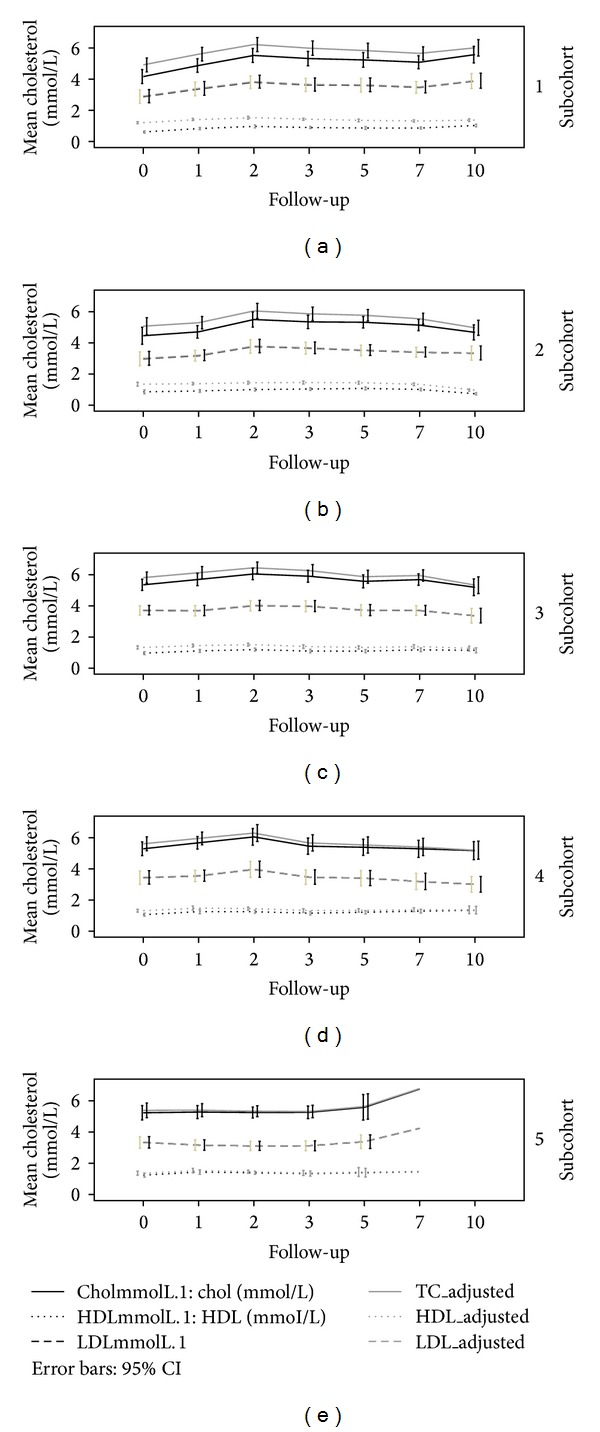
Lipoprotein levels measured over time in the various subcohorts before and after correction with the lipid decay factor. Mean TC and HLD-c levels are depicted on the *y*-axis and follow-up time on the *x*-axis.

As a significant decay effect was found for TC and HDL-c, a correction factor was estimated. As storage time increased, a decrease (95% CI) was observed, −0.03 mmol/L (−0.045–−0.015) for TC and −0.024 mmol/L (−0.027–−0.021) for HDL-c per year of increasing storage time. The decrease in HDL-c levels was relatively larger (considering the range) than the decrease in TC levels at same length of storage. The TC/HDL ratio calculated for the same sample will therefore become higher as a direct result of increasing storage time and the disproportionate decay effect on TC and HDL-c. This lipid decay was estimated to be linear. A lipid decay correction factor was calculated to be [*y* = *β*observed + (*β*Chol∗*t*)] 0.03 mmol/L for TC and 0.024 mmol/L for HDL-c.  Figure 3 illustrates the differences in the course of unadjusted and adjusted cholesterol levels.

### 3.4. Clinical Impact of the Storage Decay Effect on Lipids

The storage decay effect of lipids during storage affects the TC : HDL-c ratio. This ratio is used when calculating the 10-year CV risk of individual patients in a clinical setting. Patients are then categorized as either “low” risk (<10% 10-year risk of a CV event), “intermediate” risk (10–20% 10-year risk of a CV event), or “high” risk (>20% 10-year risk of a CV event). As a result of the storage decay effect, the TC : HDL-c ratio calculated using the measured lipoprotein levels decreases as storage time increases. To better approximate the lipoprotein levels at the time the serum sample was taken, and the lipid decay correction factor was estimated. Correction for the storage decay effect will yield a higher (improved) TC : HDL ratio which reduces the calculated CV risk. After applying the storage correction factor, the TC : HDL-c ratio decreased. Before correction, TC : HDL ratio was classified as high in 75% of patients, compared to 54% after correction (not shown). After correction, patients moved to lower CV risk groups, as the TC : HDL-c ratio decreases after correction for the storage decay effect (Table 4). Before correction, most patients were categorized in the “low” CV risk group (<10%) and 53 patients were reclassified from the intermediate and high risk groups to this group, totaling 552 patients (an 11% increase) after correction. The intermediate (10–20%) and high (>20%) CV risk groups decreased in size by 8% and 11%, respectively. Overall, in this cohort of 1050 patients, 53 (5%) patients changed CV risk groups according to SCORE 10-year risk predictions for CVD.

## 4. Discussion

Our study is the first to investigate the suitability of cholesterol levels obtained in serum samples that have been stored for more than 10 years and to evaluate if this storage decay effect is clinically important for CV risk evaluation in RA patients using available CV risk calculators. We show that a significant but modest storage decay effect on cholesterol levels occurs over time. The magnitude of this effect per additional year of storage is relatively small. Correcting factors to adjust for this effect were calculated, 0.03 mmol/L for TC and 0.024 mmol/L for HDL-c, per additional year of storage. Interestingly, the change in TC and HDL-c per additional year of storage in this cohort is similar, which means that the impact of this storage decay effect will be greater for HDL-c, as the range of HDL-c levels is much smaller compared to TC levels. The decay effect of TC levels reported in this study is less steep than the decrease in HDL-c. Therefore, the observed TC : HDL ratio may become increased and lead to false-higher CV risk predictions. In particular, lipid measurements in samples that have undergone long-term storage could be inaccurate, which would eventually lead to distorted risk predictions if these values were used in a CV risk model. However, this does not seem to be the case. Clinical impact of the storage decay effect appears to be minimal, with only a small number of patients (5%) moving from groups indicated for primary CV prevention (either the intermediate or high risk) to the low risk group after application of a correction factor. Although, on an individual level, changes in absolute lipid levels may be relevant, these changes do not appear to significantly affect cardiovascular risk predictions. Therefore, it may not be necessary to use a correction factor when samples have been stored under stable conditions.

Determining whether long-term storage affects the integrity of lipid samples requires analysis of long-term data. Ideally one would compare values determined in the past, directly after acquiring the blood sample (“original” values), with values determined from stored samples (“observed” values) within patients. The differences between “original” values and “observed” values provide an immediate indication of the storage effect. Several studies have used this approach but in serum samples that were stored for relatively short periods of 1-2 years [19, 20, 24, 25]. However, measurements of lipids directly after blood sampling are not always available in RA cohorts. This may be due to the fact that the awareness of the increased risk of CVD gained particular interest long after the start of RA cohorts. Hence a different approach is required to assess the storage decay effect on lipid levels in these cohorts. In a study that investigated the effect of long-term storage (7 years) [26], the group means of lipids for pairs of serial specimens that were taken at 6- and 12-month intervals were compared. It was assumed that in the absence of a storage effect the variation in group means would reflect only normal biological variation, which would not lead to a systematic downward or upward effect in the group mean cholesterol levels. Subsequently, any observed changes would reflect the storage effect [26]. However, in a long-running cohort that includes a lengthy follow-up of more than 10 years, patients could also be systematically different between different time-periods. For example, patients that were included more recently are probably more likely to use statins at the time the serum samples were taken than patients that were included 25 years ago. In this cohort, cholesterol levels from samples that were stored the shortest could therefore be systematically lower than samples that were stored the longest, without involvement of a storage effect. Such a period effect could lead to biased results. Therefore, we used a method in which the data were grouped according to five different time-periods for analysis, to adjust results for a period effect. Illustratively, statin use at baseline increased from 0% in the first three subcohorts (1985–1999) to 29% in the last cohort (2005–2009). The percentage of smokers decreased from 47% to 29% during that same timeframe. Overall, these subcohorts did not seem to differ significantly, excluding period effect as a confounder. Consequently, a simplified model was developed. The methodology used in this study can also be applied in other cohorts.

This study has limitations. The storage effect was estimated without knowing “original” values for comparison. The correction factor was directly derived from the regression coefficient of storage time. It was assumed that after correction for repeated measures and for confounders any observed change is attributable to the storage effect. Biological variation of lipid levels within a subject could potentially contribute to the found decay effect over time. However, several (*n* = 7) measurements per patient were included, in the total group of 152 patients. Serum samples had been stored under similar condition, albeit in different storage facilities. It was considered unlikely that any variation, either due to biological variation within patients or due to differing storage circumstances, would contribute to an overall, systemic trend. It has been previously suggested that such variability in measured cholesterol levels is unpredictable and often a wide variation in both directions (±20%) is reported [30, 31]. In the model that was formulated, the most important confounders were dealt with, but the existence of other confounding factors cannot be excluded. Storage conditions, varying temperatures during storage, and number of times the samples were thawed are all factors that could have contributed in affecting serum cholesterol levels. As multiple sets of samples from 152 patients were used, it is considered unlikely that any of these highly variable factors contributed significantly to the systemic decay effect that was found. In addition, modifying the storage temperature from −20°C to −80°C for all serum samples in 2008 is a limitation, although the vast majority of the samples (1985–2007) were stored under the same conditions. Storage effects are likely to be even smaller with lower temperatures.

In conclusion, the results of this study show that modest decay of lipid levels in serum samples should be expected during long-term (>10 years) storage. Using the method proposed here, a correction factor can be formulated to adjust for this storage decay effect. However as the clinical impact on CV predictions appears minimal, extensive adjustment may not be necessary to obtain valid lipid levels. Hence, stored serum samples, even when stored for long periods of time, are a valuable source of data. These data can be of particular importance for studies in RA cohorts involving long-term outcomes such as cardiovascular disease. In addition, it may be financially attractive to minimize the analysis period by performing cholesterol measurements in stored serum samples all at once at the end of a long-term study with multiple samples taken over long periods of time. It is recommended to employ the method presented in this study in other long-term cohorts.

## Figures and Tables

**Table 1:** Patient characteristics.

*n* = 152	Subcohorts				
	1 (*n* = 30) 1985–1989	2 (*n* = 31) 1990–1994	3 (*n* = 30) 1995–1999	4 (*n* = 30) 2000–2004	5 (*n* = 31) 2005–2009
Age^a^	51 ± 14.4	50 ± 13.5	52 ± 14.5	58 ± 12.1	59 ± 12.6
Female^b^	16 (53.3)	19 (61.3)	17 (56.7)	25 (83.3)	21 (67.7)
RF positive^b^	26 (86.7)	23 (74.2)	25 (83.3)	23 (76.7)	25 (80.6)
DAS28^a^	5.6 ± 1.2	5.3 ± 1.4	4.8 ± 1.6	4.9 ± 1.0	5.0 ± 1.2
BMI^a^	26 ± 3.8	27.1 ± 4.6	25.6 ± 3.3	26.2 ± 3.0	26.6 ± 6.8
Smokers^b^	14 (46.7)	6 (19.4)	10 (33.3)	9 (30.0)	9 (29.0)
Statin use^b^	0 (0.0)	0 (0.0)	2 (6.7)	2 (6.7)	9 (29.0)
Glucocorticosteroid use^b^	3 (10.0)	3 (9.7)	5 (16.7)	17 (56.7)	17 (54.8)
Baseline lipoprotein levels					
TC (mmol/L)^a^	4.2 ± 1.2	4.5 ± 1.5	5.4 ± 1.0	5.3 ± 1.2	5.2 ± 1.2
HDL-c (mmol/L)^a^	0.6 ± 0.2	0.9 ± 0.3	1.0 ± 0.3	1.1 ± 0.2	1.2 ± 0.4
TC : HDL-c^a^	7.2 ± 1.9	5.5 ± 1.6	5.9 ± 1.4	5.1 ± 1.2	4.5 ± 1.2
Treatment during follow-up					
B-DMARDS^b^	9 (30.0)	13 (41.9)	10 (33.3)	17 (56.7)	3 (9.7)

(i) Abbreviations: RF, rheumatoid factor; DAS28; disease activity index 28 joints, BMI: body mass index, TC, total cholesterol; HDL-c, high-density-lipoprotein cholesterol, B-DMARDS; biological disease modifying anti-rheumatic drugs.

(ii) ^a^Data presented as mean ± SD, ^b^Data presented as *n* (%).

**Table 2:** Effect of storage time (years) on TC levels.

	Estimate	SE	*P-value *	95% CI	
				Lower	Upper
Constant	3.408	0.389	<0.0001	2.65	4.17
Storage time	−0.030	0.008	<0.0001	−0.045	−0.015
Time within patients (follow-up)	0.012	0.003	<0.0001	0.001	0.018
Time^2^ (follow-up)	−0.0001	0.00003	<0.0001	−0.0002	−0.0001
Gender	0.023	0.097	0.810	−0.167	−0.213
Age	0.016	0.004	<0.0001	0.087	0.023
Statin use at baseline	0.888	0.188	<0.0001	0.519	1.256
BMI	0.011	0.011	0.343	−0.011	0.033
Glucocorticosteroid use at baseline	0.239	0.115	0.038	0.013	0.465

(i) SE, standard error; BMI, body mass index; and 95% CI: 95% confidence interval.

(ii) Data are adjusted for age, gender, statin use at baseline, BMI, and glucocorticosteroid use at baseline.

(iii) Time^2^ is a quadratic term that was included due to the nonlinear course of cholesterol levels over follow-up time, and this variable also represents time within patients (follow-up time).

**Table 3:** Effect of storage time (years) on HDL-c levels, adjusted results.

	Estimate	SE	*Pvalue *	95% CI	
				Lower	Upper
Constant	1.247	0.056	<0.0001	1.136	1.358
Storage time	−0.024	0.002	<0.0001	−0.027	−0.021
Time within patients (follow-up)	0.003	0.001	<0.0001	0.001	0.004
Time within patients^2^ (follow-up)	−0.00003	0.00001	<0.0001	−0.00004	−0.00002
Age	0.003	0.001	0.001	0.001	0.004
Gender	−0.074	0.021	0.001	−0.117	−0.031

(i) SE, standard error; 95% CI, 95% confidence interval.

(ii) Data are adjusted for age, gender, and time within patients^2^.

**Table 4:** CV risk reclassification.

CV risk	Before correction for storage decay *n* (%)	After correction for storage decay *n* (%)	Reclassified *N* = 53
Low (<10%)	499 (48%)	552 (53%)	+53 (11%)
Intermediate (10–20%)	178 (17%)	164 (16%)	−14 (8%)
High (>20%)	373 (35%)	334 (31%)	−39 (11%)

Total *n* (%)	1050 (100%)	1050 (100%)	+53 (5%)

(i) CV risk, cardiovascular risk.

(ii) Number of patients reclassified according to the SCORE CV risk categories for the 10-year risk of a CV event; (low; <10%, intermediate; 10–20%, and high; >20%) before and after correction for storage decay effect on TC and HDL-c.

## Abbreviations

CV: Cardiovascular
SCORE: Systematic coronary risk evaluation
TC: Total cholesterol
HDL-c: High density lipoprotein cholesterol
TG: Triglycerids
DMARD: Disease-modifying antirheumatic drug
LDL-c: Low-density lipoprotein cholesterol
RF: Rheumatoid factor
TC : HDL: Total cholesterol : high density lipoprotein cholesterol ratio.
